# COVID-19 disruptions and spring surges: an interrupted time-series analysis of tuberculosis diagnostic challenges in Northeast Iran

**DOI:** 10.1038/s41598-026-45863-w

**Published:** 2026-05-02

**Authors:** Nafiseh Izadi, Faranak Alizadeh, Zahra Meshkat, kiarash Ghazvini, Shaho Menbari, Ehsan Aryan

**Affiliations:** 1 Antimicrobial Resistance Research Center, Basic Sciences Research Institute, Mashhad University of Medical Sciences, Mashhad, Iran; 2 Department of Microbiology and Virology, Faculty of Medicine, Mashhad University of Medical Sciences, Mashhad, Iran; 3 Department of Medical Laboratory Sciences, Faculty of Paramedical, Kurdistan University of Medical Sciences, Sanandaj, Iran

**Keywords:** *Mycobacterium tuberculosis*, Tuberculosis epidemiology, Seasonal trends, Diagnostic discordance, COVID-19 impact, Iran, Public health, Diseases, Health care, Medical research, Microbiology

## Abstract

Tuberculosis (TB) remains a significant public health challenge in Northeast Iran, yet longitudinal data evaluating regional transmission patterns following COVID-19 disruptions remain limited. This cross-sectional study, conducted from 2017 to 2023 at Qaem University Hospital, Mashhad, Iran, analyzed 14,572 patients with suspected TB using smear microscopy, culture, and PCR to characterize the test positivity rate (TPR), temporal shifts, and diagnostic challenges. We identified a 10.3% TPR (1,494 out of 14,572), with significant demographic disparities: females accounted for 51.7\% of cases (OR = 0.74, 95\% CI: 0.665–0.824; *p* < 0.001), although the association strength was weak (Cramér’s *V* = 0.05). Adults aged 65 and older represented 51.6\% of the cases. The COVID-19 pandemic led to a 33.3\% decline in diagnoses from 2019 to 2020, with outpatient recovery lagging behind inpatient services. Time-series analysis identified a significant structural break in March 2020 (*p* < 0.001), statistically confirming the sharp decline in diagnoses due to the pandemic. Bronchoalveolar lavage showed the highest positivity rate at 54.7\%, identifying 135 smear-negative/culture-positive cases. Seasonal peaks in spring (27.8\%) are hypothesized to result from post-winter Vitamin D troughs and social gatherings during Nowruz. These findings emphasize the importance of geriatric-focused screening, multimodal diagnostic protocols, and pandemic-resilient TB surveillance. Regional policies should focus on integrated respiratory screening and community-based interventions to reduce seasonal transmission.

## Introduction

Tuberculosis (TB) is an infectious disease primarily caused by *Mycobacterium tuberculosis*, affecting the lungs (pulmonary TB) and potentially other organs (extrapulmonary TB)^1^. Despite advances in diagnostics and treatment, TB remains a significant public health issue, especially in regions with high disease burden and increasing drug-resistant strains^2^. The interaction between TB and the HIV/AIDS epidemic has led to an increase in its incidence in many countries. Following the COVID-19 pandemic, TB became the second leading cause of death from infectious diseases worldwide^3^. In 2022, about 10.6 million new TB cases were reported, leading to an estimated 1.3 million deaths, mainly in middle- and low-income countries where factors like poverty, malnutrition, and overcrowding worsen transmission. In Iran, TB incidence is classified as intermediate, with approximately 13 cases per 100,000 people according to recent estimates, underscoring the need for ongoing public health efforts to tackle this disease^4, 5, 6^.

The 2023 WHO Global Tuberculosis Report (which presents data collected during the 2022 calendar year) highlights neighboring countries like Azerbaijan, Turkmenistan, Armenia, Pakistan, Afghanistan, and Iraq as high-burden TB nations, each with incidence rates over 20 cases per 100,000 population^4^. Iran’s extensive borders with these countries have led to increased immigration and travel, complicating efforts to control the disease^5^. Moreover, TB distribution across Iran is heterogeneous, with high incidence in border regions such as Sistan and Baluchestan, Golestan, Khorasan, South Azerbaijan, West Azerbaijan, and Kurdistan. Particularly in Khorasan, which has the third highest incidence after Sistan and Baluchestan and Golestan^7, 8, 9, 10^. This geographic disparity necessitates targeted public health interventions for these vulnerable areas.

The frequency of TB-positive cases can vary based on diagnostic methods, influenced by the clinical suspicion threshold, sample quality, lab performance, resource availability, and adherence to testing guidelines^11^ As a result, rigorous monitoring and evaluation of TB diagnostic accuracy are essential. Incomplete reporting of positive TB cases in some regions undermines monitoring and control efforts, prompting the WHO to call for comprehensive studies to track TB transmission patterns and inform public health strategies.

Filling this scientific gap, this study evaluates the Test Positivity Rate (TPR) and diagnostic yield of active TB among patients referring to Qaem University Hospital in Mashhad, Iran. As a leading tertiary referral center, this hospital serves as a representative site for identifying complex and paucibacillary cases in the region. By analyzing the demographic and clinical characteristics of TB-positive cases, the research aims to provide insights into the current epidemiological landscape of TB in the region, contributing to a better understanding of the disease and informing public health strategies to reduce its transmission.

## Materials and methods

### Study design and setting

This cross-sectional study was conducted at Qaem University Hospital, Mashhad, Iran, from 2017 to 2023. A total of 14,572 patients presenting with presumptive tuberculosis (TB) symptoms were enrolled. In accordance with the Iranian National Tuberculosis Control Program, a suspected (presumptive) case was defined as any individual presenting with a persistent productive cough lasting > 2 weeks, or those with constitutional symptoms (e.g., evening fever, night sweats, or unexplained weight loss), or individuals with documented close contact with a smear-positive pulmonary TB case. Inclusion criteria required all referred symptomatic patients (both outpatients, 55.2\%, *n* = 8,038; and inpatients, 44.8\%, *n* = 6,534) who provided informed consent. Exclusion criteria were clinical specimens of inadequate quality (e.g., salivary samples or volume < 3 mL), patients who had received anti-TB treatment within the preceding 6 months to avoid false-positive PCR results from residual DNA, and cases with incomplete clinical records. Ethical approval for the study was granted by the Institutional Review Board of Mashhad University of Medical Sciences (Approval Code: IR.MUMS.MEDICAL.REC.1402.419), and written informed consent was obtained from all participants.

### Sample collection and processing

Clinical specimens, including bronchoalveolar lavage (BAL), sputum, pleural fluid, cerebrospinal fluid (CSF), and tissue biopsies, were collected aseptically. Samples were transported in sterile, leak-proof containers to the laboratory within 2 h of collection. Sputum and BAL samples were homogenized with an equal volume of 1% N-acetyl-L-cysteine (Sigma-Aldrich, USA) and decontaminated using the standard NaOH-NALC method. Processed samples were centrifuged at 3,000 × *g* for 15 min (Eppendorf Centrifuge 5804R, Germany), and the sediment was resuspended in 2 mL phosphate-buffered saline (PBS, pH 7.2) for downstream testing.

### Microbiological testing

A case was considered laboratory-confirmed if it met at least one of the following criteria: identification of acid-fast bacilli (AFB) via smear microscopy, isolation of *M. tuberculosis* complex via culture, or detection of specific genomic markers via PCR.

#### Smear microscopy

Smears were prepared from the resuspended sediment, fixed with heat, and stained using the Ziehl-Neelsen method. The slides were examined under a light microscope (Olympus CX23, Japan) at 100× magnification. Acid-fast bacilli (AFB) were quantified according to the World Health Organization (WHO) grading system.

#### Culture

For mycobacterial culture, 500 µL of processed sample was inoculated onto Löwenstein-Jensen (LJ) slants (Merck, Germany) and incubated at 37 °C. Cultures were monitored weekly for growth over 8 weeks.

#### Molecular testing

DNA extraction was performed using the boiling method^12^. Polymerase chain reaction (PCR) targeting the *hsp65* and IS*6110* genes was conducted with 500 nM of the following primers:

*hsp65* gene: TB 11/12 (5′-ACCACCGATGGTGTGTCCAT-3′ / 5′-GTTGTCGAACCGCATACCCT-3′)^13^.

*IS6110* gene: INS 1/2 (5′-CGTGAGGGCATCGAGGTGGC-3′ / 5′-GCGTAGGCGTCGGTGACAAA-3′)^14^.

The PCR was performed with the following thermal cycling conditions: initial denaturation at 95 °C for 5 min, followed by 35 cycles for the *hsp65* gene at 95 °C for 30 s, 60 °C for 30 s, and 72 °C for 30 s. For the IS*6110* gene, the conditions were 95 °C for 1 min, 65 °C for 1 min, and 72 °C for 1 min. The amplification was completed with a final extension at 72 °C for 5 min.

### Statistical analysis

Data were analyzed using SPSS version 22.0 (IBM, USA) and GraphPad Prism 8.0 (GraphPad Software, USA). Descriptive statistics were reported as frequencies and percentages. Associations between categorical variables (e.g., gender, age, season) and TB positivity (Test Positivity Rate) were assessed using Pearson’s chi-square test. The strength of associations was quantified using Cramér’s V, interpreted as: 0.00–0.10: Weak association; 0.10–0.30: Moderate association; 0.30–0.50: Strong association; 0.50 and above: Very strong association. Logistic regression models were employed to estimate odds ratios (OR) with 95% confidence intervals (CI). A *p*-value < 0.05 was considered statistically significant.

Furthermore, to statistically evaluate the impact of the COVID-19 pandemic on TB case detection trends, an Interrupted Time Series (ITS) analysis was conducted. We employed the Auto-Regressive Integrated Moving Average (ARIMA) model using the SPSS Expert Modeler. The model was configured to automatically detect structural breaks and outliers (additive, level shift, or transient) to quantify the magnitude and significance of disruptions in the time series data. Model fit was assessed using the Ljung-Box Q statistic and stationary R-squared values.

The study adhered to the Declaration of Helsinki. The ethics committee of Mashhad University of Medical Sciences approved all procedures involving human participants. Confidentiality of patient data was maintained through anonymized identifiers.

## Results

### Demographic and clinical characteristics

**Fig. 1:**
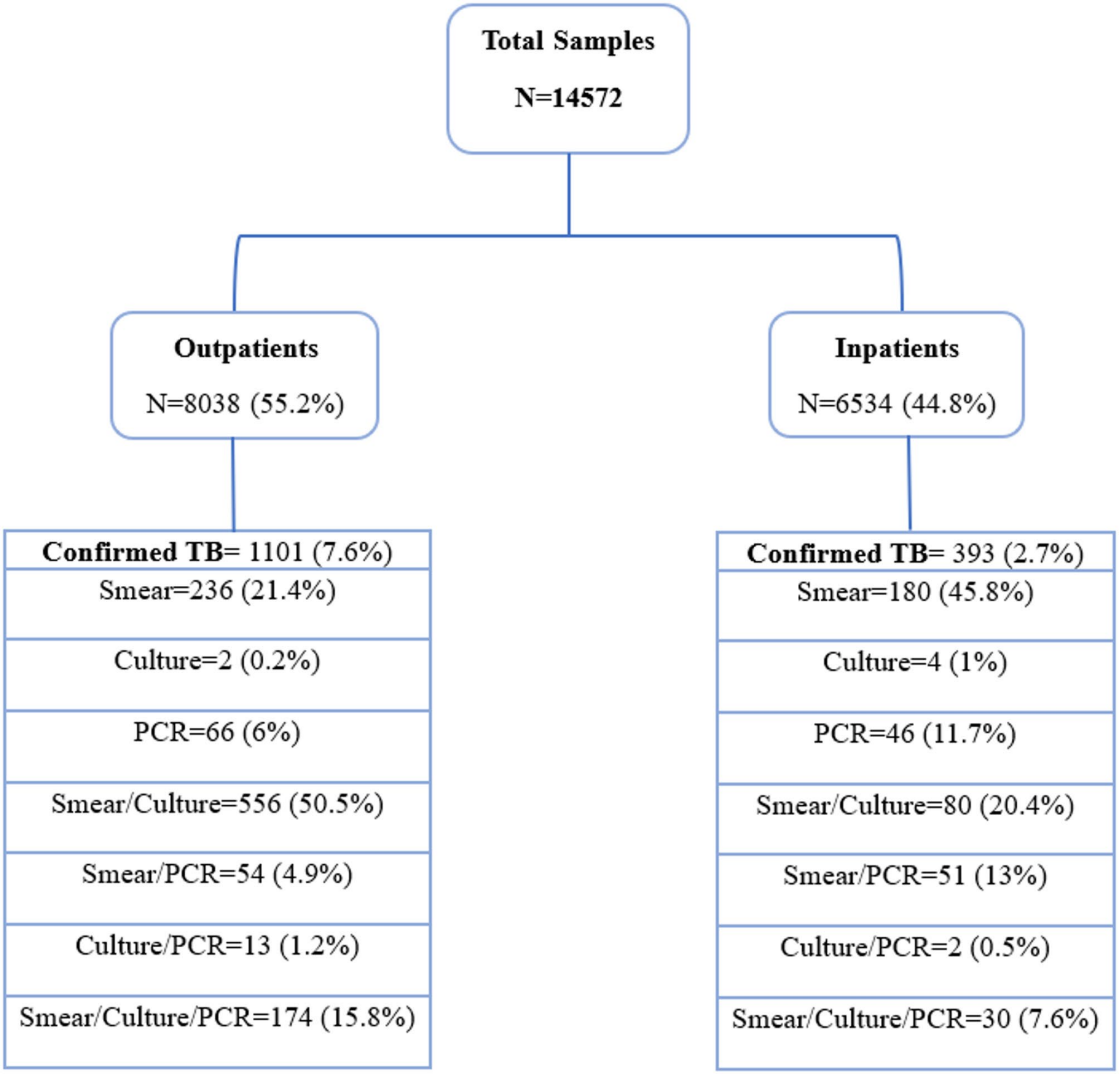
Overview of study samples from Qaem Hospital (2017–2023): A total of 14,572 patients were screened for TB infection, categorized into outpatients (8,038; 55.2%) and inpatients (6,534; 44.8%). The confirmed TB cases are presented by diagnostic method, highlighting the prevalence of infection within each subgroup and offering insights into the effectiveness of various diagnostic approaches.

Among 14,572 patients screened for tuberculosis (TB) at Qaem Hospital from 2017 to 2023, a total of 1,494 (10.3%) tested positive, representing the overall Test Positivity Rate (TPR) or diagnostic yield within this clinical cohort (Fig. 1). Females accounted for a higher proportion of cases (51.7%, *n* = 773) compared to males (48.3%, *n* = 721). While this association was statistically significant (χ² (1) = 30.52, *p* < 0.001), the effect size was very weak (Cramér’s *V* = 0.05, 95% CI [0.030, 0.062]), suggesting that gender is a minor predictor of TB positivity in this setting. Logistic regression confirmed males had 26% lower odds of testing positive (OR = 0.74, 95% CI [0.665, 0.824]).

**Fig. 2:**
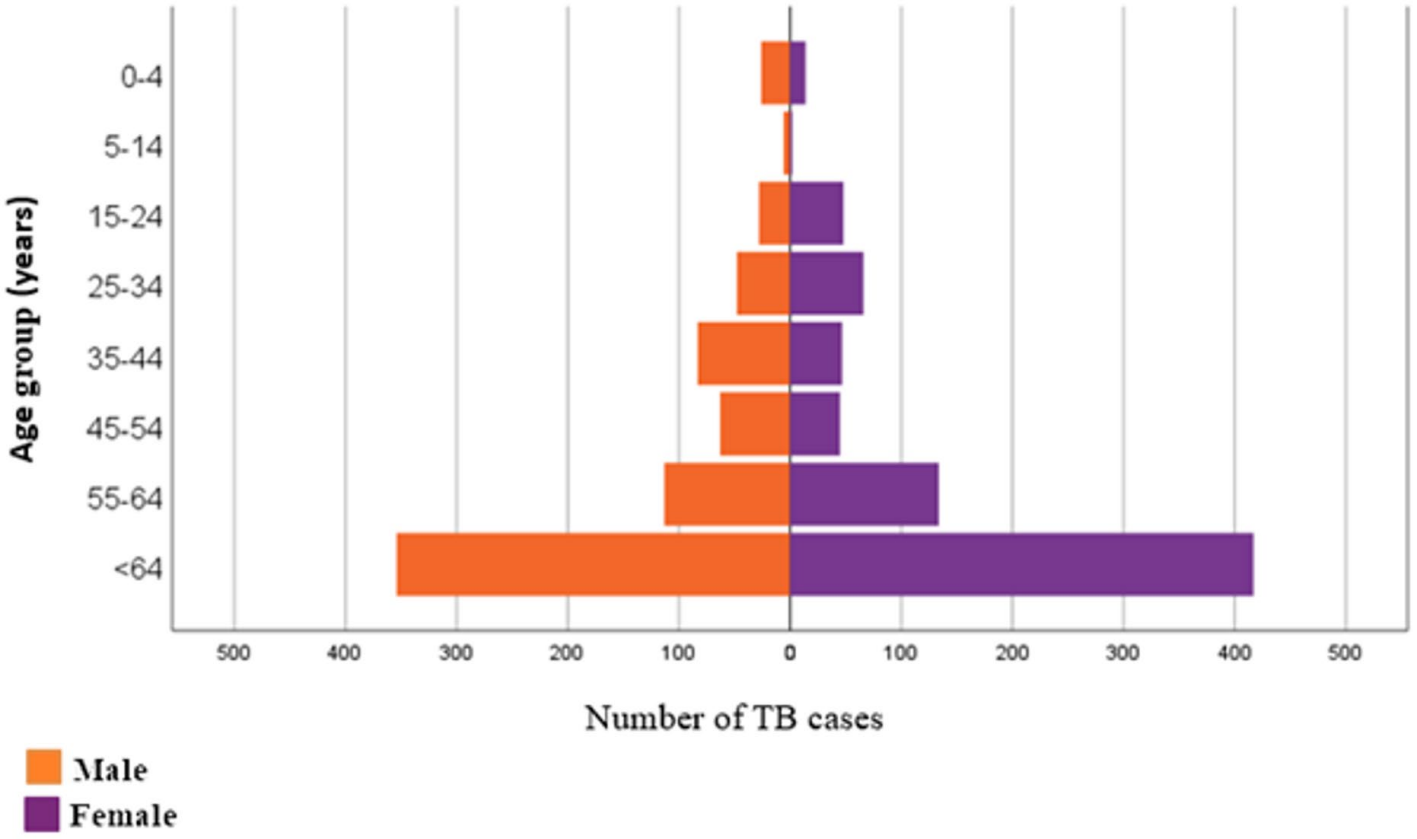
Distribution of tuberculosis cases by age group and gender at Qaem Hospital, Mashhad, Iran (2017–2023).

**Fig. 3:**
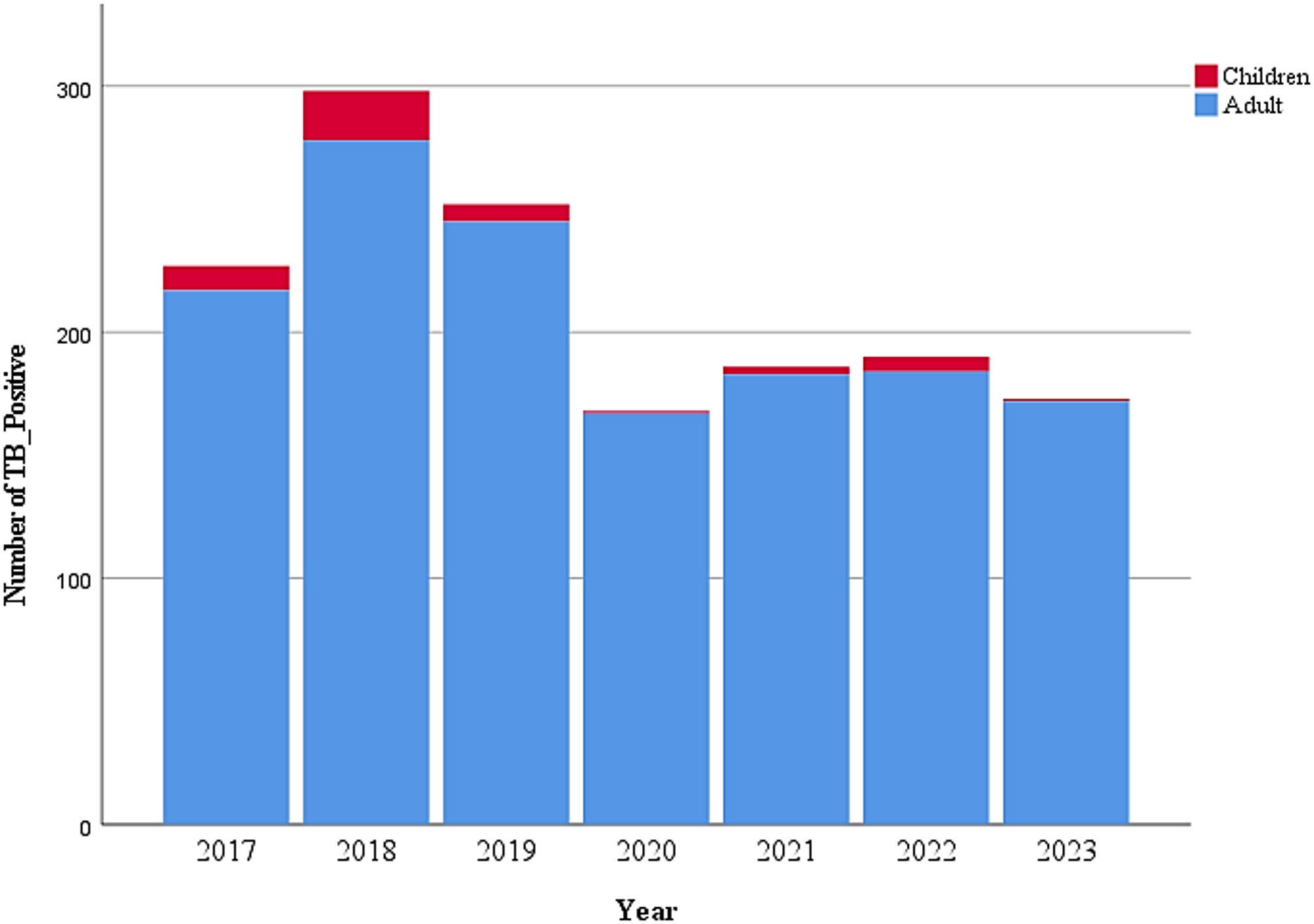
Annual distribution of TB-positive cases by year and age group (Children vs. Adults) at Qaem Hospital, Mashhad, Iran (2017–2023).

Outpatients constituted 73.7% (*n* = 1,101) of TB-positive cases, showing 2.37 times higher odds of positivity than inpatients (OR = 2.37, 95% CI [2.097, 2.676]; Cramér’s *V* = 0.12, *p* < 0.001). The age distribution revealed a significant burden among older adults, with 51.6% (*n* = 771) of cases occurring in individuals aged ≥ 65 years, followed by the 55–64 age group (16.5%, *n* = 247). Children (0–14 years) accounted for only 3% (*n* = 45) of cases (Figs. 2 and 3). A weak association between age and TPR was observed (Cramér’s *V* = 0.09, *p* < 0.001; 95% CI [0.080, 0.108]) (Table 1).

### Diagnostic methods and discordant results

A combination of smear microscopy, culture, and PCR testing was used for diagnosis (Fig. 1). Outpatients mainly underwent combined smear and culture testing (*n* = 4,313), resulting in 556 positive cases, while inpatients were primarily screened using smear microscopy (*n* = 3,624), with 180 positives. There were notable discrepancies in the results obtained from different diagnostic methods (Table 2), including 135 cases with smear-negative but culture-positive results, and 17 cases (1.1% of positives) that were negative by both smear and culture but confirmed positive by PCR. Additionally, 27 cases showed conflicting results across all three methods.

### Specimen-specific positivity rates

Bronchoalveolar lavage (BAL) specimens accounted for 47.7% (*n* = 6,648) of collected samples and achieved the highest diagnostic yield (54.7%, *n* = 780/1,427 of total positives), significantly outperforming sputum (39.3% positivity among *n* = 5,369 sputum samples). Extrapulmonary specimens, such as pleural fluid (1.4% positivity) and cerebrospinal fluid (0.8%), had substantially lower yields (Table 3). A significant association between specimen type and TB results was observed (χ²(8, *N* = 13,947) = 96.73, *p* < 0.001), although the effect size was weak (Cramér’s *V* = 0.083, 95% CI [0.000, < 0.001]).

### Temporal shifts and COVID-19 impact

**Fig. 4:**
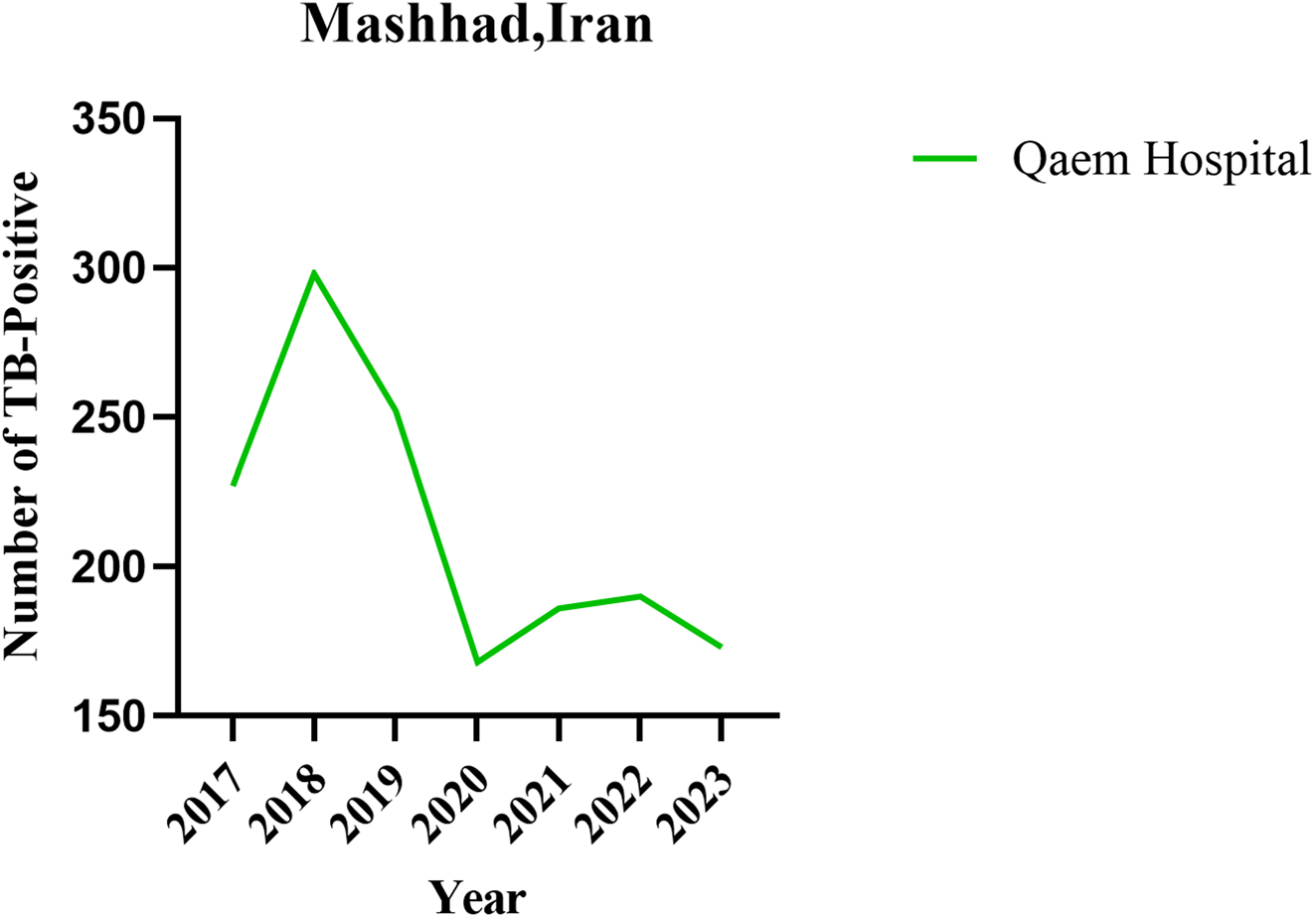
Temporal Shifts in TB-Positive Cases at Qaem Hospital, Mashhad, Iran (2017–2023). The sharp decline observed in 2020 corresponds to a significant structural break (transient outlier) statistically confirmed by ARIMA time-series modeling (*p* < 0.001), reflecting the impact of the COVID-19 pandemic.

Analysis of annual distributions showed a 33.3% decrease in laboratory-confirmed TB diagnoses from 2019 (*n* = 252) to 2020 (*n* = 168), coinciding with the onset of the COVID-19 pandemic (Fig. 4). The diagnostic positivity rate fluctuated over time, reflecting pandemic-related healthcare disruptions. Outpatient cases decreased significantly (from approximately 200 to 130), while inpatient declines were less pronounced (from around 50 to 30). Partial recovery in case detection occurred in 2021 (*n* = 186) and 2022 (*n* = 190), followed by a decrease in 2023 (*n* = 173), indicating ongoing post-pandemic challenges in regional TB surveillance.

Time-series modeling (ARIMA) further confirmed the significant seasonality of TB transmission in the region [Model: ARIMA(0,1,1)(1,0,0); Seasonal AR parameter = 0.396, *p* = 0.001]. Most importantly, the outlier detection algorithm statistically identified a significant Transient Outlier in March 2020, coinciding exactly with the onset of the COVID-19 outbreak in Iran. The model estimated a sharp reduction of approximately 123 cases in that month compared to the expected trend (Magnitude = -122.80, t = -5.024, *p* < 0.001). This finding provides robust statistical evidence of a structural break in case detection due to pandemic-related disruptions.

### Hospital admission status

**Fig. 5:**
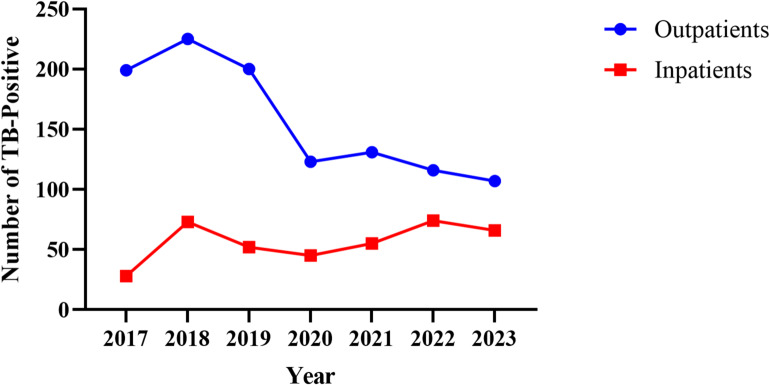
Temporal Trends in TB-Positive Cases by Hospital Admission Status at Qaem Hospital, Mashhad, Iran (2017–2023).

Outpatient TB cases declined sharply during the pandemic (from ~ 200 to 130) but partially recovered by 2022 (*n* = 160). Inpatient cases showed a more minor reduction (from ~ 50 to 30) (Fig. 5).

### Seasonal and monthly variations

**Fig. 6:**
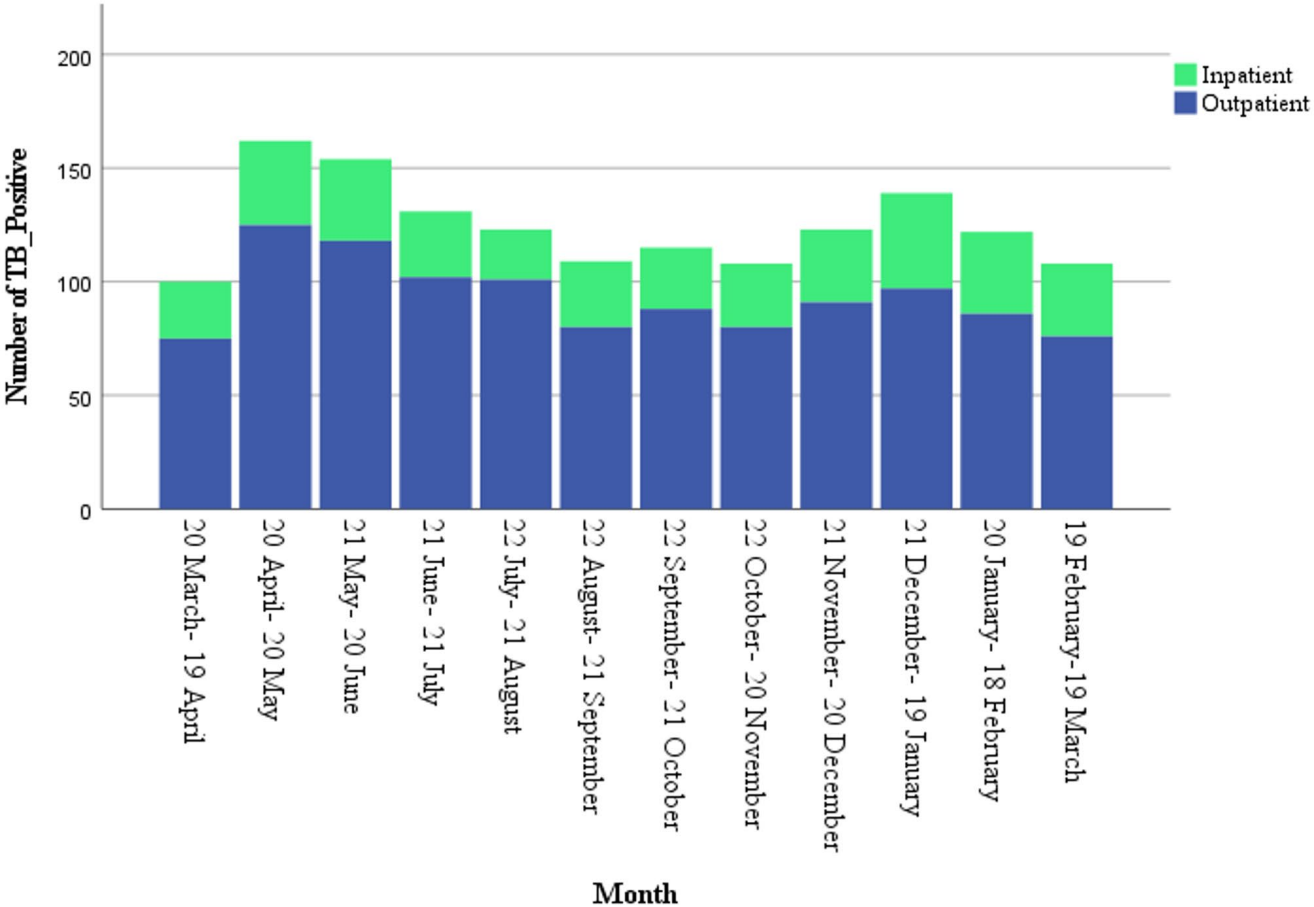
Monthly Distribution of TB-Positive Cases by Hospital Admission Status at Qaem Hospital, Mashhad, Iran (2017–2023).

**Fig. 7:**
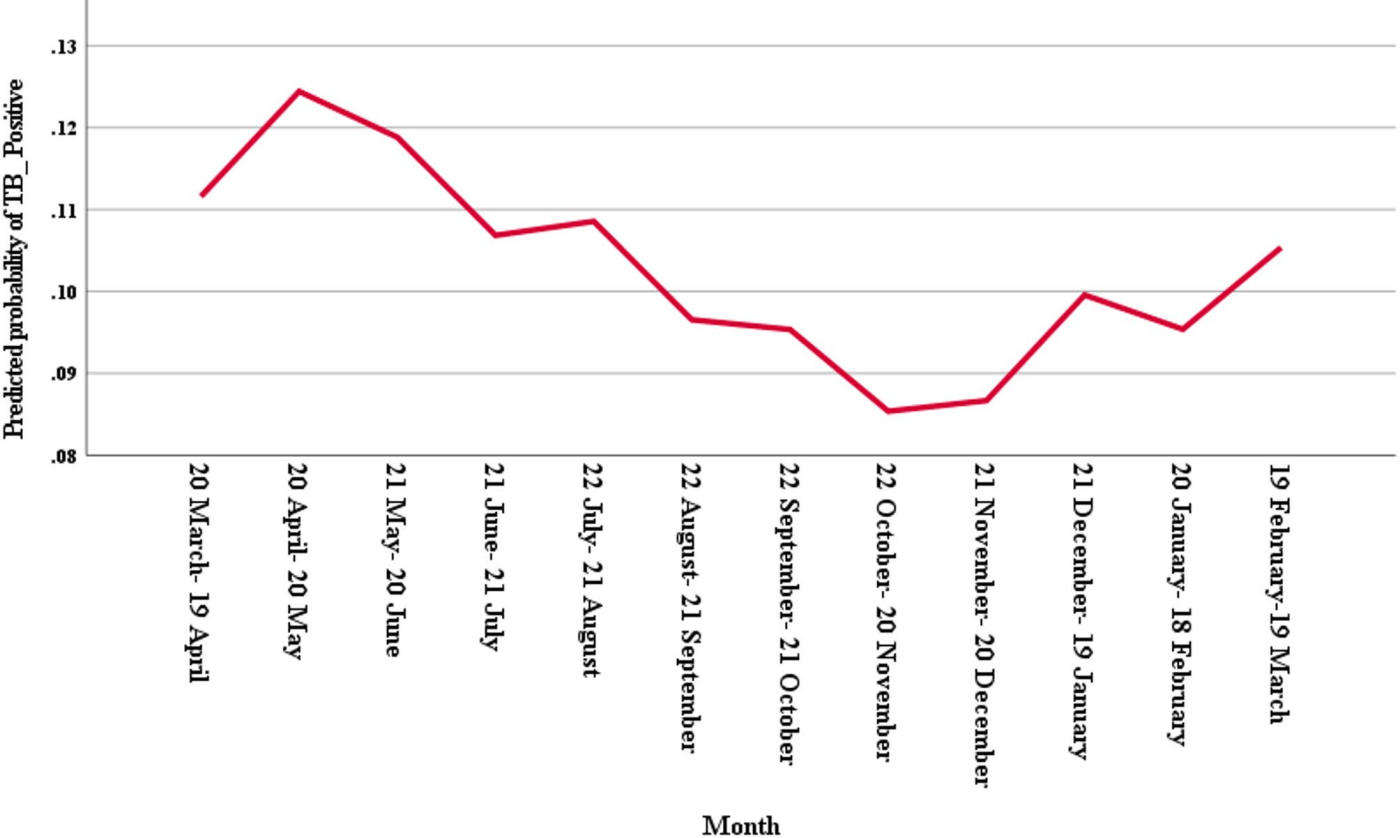
Predicted Probability of TB Positivity by Month at Qaem Hospital, Mashhad, Iran (2017–2023).

Seasonal distribution analysis revealed a peak in spring (27.8%, *n* = 412 of positive cases), with the highest monthly positivity observed between 20 April and 20 May (11.2%, *n* = 166; Fig. 6). Autumn had the lowest positivity rate (23.2%, *n* = 346) but the highest proportion of TB-negative suspects (27.1%). Multivariate logistic regression analysis showed significantly lower odds of a positive result during non-spring seasons compared to spring: Summer (OR = 0.859, 95% CI [0.740, 0.998]), Autumn (OR = 0.722, 95% CI [0.621, 0.840]), and Winter (OR = 0.820, 95% CI [0.707, 0.951]). Monthly interval analysis (Fig. 7) reveals temporal patterns, with peak predicted probabilities of TB positivity between 20 April and 20 May (probability = 0.12) and a nadir during autumn (22 September–21 October; probability = 0.09).

A statistically significant but modest monthly association was detected (Cramér’s V = 0.04, χ²(11) = 21.99, *p* = 0.02; Wald χ² = 21.870, *p* = 0.025), indicating seasonal variability persists at marginal significance thresholds.

**Table 1:** Distribution of TB-Positive cases by gender, Hospital admission, age categories, season, and month from 2017 to 2023 at Qaem Hospital in Mashhad, Iran.

		Data analysis (*N* = 1494)				
		N (%)	Cramer’s V	*P value*	95% Confidence Interval	
					Lower	Upper
Gender	Male	720 (48.2%)	0.05	0.001	0.030	0.062
	Female	775 (51.8%)				
Hospital admission	Outpatient	1101 (73.7%)	0.12	0.001	0.103	0.132
	Inpatient	393 (26.3%)				
Age groups	0–4	41 (2.7%)	0.09	0.001	0.080	0.108
	5–14	12 (0.8%)				
	15–24	77 (5.2%)				
	25–34	110 (7.4%)				
	35–44	124 (8.3%)				
	45–54	121 (8.1%)				
	55–64	259 (17.3%)				
	< 64	751 (50.2%)				
Year	2017	227 (15.2%)	0.031	0.032	0.021	0.051
	2018	298 (19.9%)				
	2019	252 (16.9%)				
	2020	168 (11.2%)				
	2021	186 (12.4%)				
	2022	190 (12.7%)				
	2023	173 (11.6%)				
Season	Spring	412 (27.6%)	0.04	0.001	0.021	0.054
	Summer	374 (25.0%)				
	Autumn	346 (23.2%)				
	Winter	362 (24.2%)				
Month	20 March- 19 April	92 (6.2%)	0.04	0.02	0.03	0.06
	20 April- 20 May	166 (11.2%)				
	21 May- 20 June	153 (10.2%)				
	21 June- 21 July	131 (8.8%)				
	22 July- 21 August	125 (8.4%)				
	22 August- 21 September	118 (7.9%)				
	22 September- 21 October	119 (8.0%)				
	22 October- 20 November	107 (7.2%)				
	21 November- 20 December	121 (8.1%)				
	21 December- 19 January	140 (9.4%)				
	20 January- 18 February	113 (7.6%)				
	19 February-19 March	109 (7.3%)				

**Cramér’s V**: 0.00–0.10: Weak association; 0.10–0.30: Moderate association; 0.30–0.50: Strong association; 0.50 and above: Very strong association.

**Table 2:** Contradictory Results of Diagnostic Methods for Tuberculosis.

Test	Smear/Culture		Smear/PCR		Culture/PCR		Smear/Culture/PCR					
Result	+/-	-/+	+/-	-/+	+/-	-/+	-/-/+	-/+/-	+/-/-	+/+/-	+/-/+	-/+/+
Number	**71**	135	**20**	**17**	10	2	**17**	**27**	**5**	**17**	**10**	**5**

**Table 3:** Clinical Samples from Qaem Hospital and TB Results, Mashhad, Iran (2017–2023).

Clinical samples of Qaem Hospital					
TB Result Specimen			Negative	Positive	Total
Pulmonary 87.2%	Bronchial lavage	N	5868	780	6648
		%	46.9%	54.7%	47.7%
	Sputum	N	4808	561	5369
		%	38.4%	39.3%	38.5%
	Gastric juice	N	127	6	133
		%	1.0%	0.4%	1.0%
Extrapulmonary 12.8%	Pleural fluid	N	494	20	514
		%	3.9%	1.4%	3.7%
	Pericardial fluid	N	32	1	33
		%	0.3%	0.1%	0.2%
	CSF	N	435	12	447
		%	3.5%	0.8%	3.2%
	Synovial fluid	N	45	1	46
		%	0.4%	0.1%	0.3%
	Ascites fluid	N	14	0	14
		%	0.1%	0.0%	0.1%
	Tissue	N	172	20	192
		%	1.4%	1.4%	1.4%
	Urine	N	72	1	73
		%	0.6%	0.1%	0.5%
	Wound	N	9	0	9
		%	0.1%	0.0%	0.1%
	Other fluids and secretions	N	443	25	468
		%	3.5%	1.8%	3.4%
	Abscess	N	1	0	1
		%	0.0%	0.0%	0.0%
Total			12,520	1427	13,947

Cramér’s V = 0.083 (Weak) 95% CI [0.000, <0.001], *p* = 0.000.

## Discussion

Tuberculosis (TB) remains a major public health concern worldwide, requiring strong surveillance systems and targeted strategies to address its complex epidemiology. Investigating the Test Positivity Rate (TPR) and diagnostic yield of TB is crucial for understanding transmission patterns within clinical settings and identifying high-risk populations. The COVID-19 pandemic has further complicated the diagnosis and treatment of TB, emphasizing the need to analyze temporal shifts in TB detection to guide policy and intervention efforts to combat current outbreaks and prepare for future health emergencies.

This cross-sectional study provides insights into the epidemiological distributions of tuberculosis (TB) in Mashhad, Northeast Iran, from 2017 to 2023. Key findings include a 10.3% TPR among symptomatic suspects, with significant demographic disparities (higher rates in females and adults ≥ 65 years), a notable decline in diagnoses during the COVID-19 pandemic (33.3% drop from 2019 to 2020), and seasonal peaks in spring (27.8% of cases). Bronchoalveolar lavage (BAL) was found to be the most effective diagnostic specimen (54.7% positivity), while discrepancies between smear, culture, and PCR results highlighted limitations in conventional diagnostic methods. These findings address the study’s primary aim of elucidating TB diagnostic dynamics in a region with unique transit and religious status and underscore the interplay of biological, environmental, and healthcare system factors in shaping TB epidemiology. The observed 10.3% TPR at our referral center is distinct from population-level prevalence; it reflects the diagnostic yield within a high-risk symptomatic cohort, which remains high compared to the national estimated incidence of 13 per 100,000 population. The higher TPR among females (51.7%), in contrast to global trends where males typically dominate TB statistics^4^, may be attributed to gendered health-seeking behaviors. Iranian women often prioritize healthcare access due to their cultural roles in family health management^5^. Additionally, it is hypothesized that biological factors such as Vitamin D deficiency, prevalent in Iranian women due to veiling practices and limited sun exposure, may contribute to increasing susceptibility^15–18^. For instance, Mishal (2001) found that veiled women in Jordan had significantly lower Vitamin D levels compared to non-veiled counterparts, a trend also observed in Bangladeshi and Japanese cohorts^16^. However, it is important to note that while the association between gender and TB positivity was statistically significant (*p* < 0.001), the effect size was very weak (Cramér’s V = 0.05), suggesting that gender is a minor predictor in this setting and these biological associations remain speculative.

The disproportionate burden among older adults (51.6% in ≥ 65 years) aligns with global trends linking age-related immunosuppression to TB risk^4^. Conversely, the low incidence among children (3%) highlights the success of Iran’s BCG vaccination programs, consistent with the country’s intermediate TB incidence classification (13 cases/100,000)^4, 6^. Notably, as this study relied on a retrospective laboratory registry, we were unable to adjust for individual-level clinical confounders such as diabetes mellitus, HIV status, or smoking history. These comorbidities are known to increase TB risk in the Iranian population, and their absence from our model necessitates caution in generalizing demographic associations. Nevertheless, the concentration of cases in the elderly underscores the urgent need for geriatric-focused screening initiatives.

The 33.3% decrease in TB diagnoses from 2019 to 2020 reflects the global disruptions caused by COVID-19, compounded by local mitigation measures in Mashhad, where Qaem Hospital was designated as a primary COVID-19 referral hub^3^. Our ARIMA time-series analysis statistically validated this disruption, identifying a significant structural break (transient outlier) in March 2020, rather than a gradual decline. Notably, outpatient diagnoses showed a slower recovery post-2020 compared to inpatient cases (Fig. 5), highlighting vulnerabilities in ambulatory care systems due to lockdowns and clinic repurposing. This is consistent with WHO reports of delayed TB notifications where pandemic-induced backlogs persisted into 2022^4^. The continued decline in 2023 diagnoses indicates health-systemic strain, including reduced testing capacity and patient reluctance—a phenomenon observed in low-income settings during health emergencies^3^. These findings emphasize the need for pandemic preparedness frameworks that prioritize TB service continuity, such as decentralized testing and telemedicine integration.

Bronchoalveolar lavage (BAL) was found to be the most effective diagnostic specimen with a positivity rate of 54.7%, surpassing sputum (39.3%). In a tertiary referral setting like Qaem Hospital, the reliance on BAL is justified for geriatric or paucibacillary patients who cannot produce high-quality sputum. However, the weak association between specimen type and TB positivity (Cramér’s V = 0.083) suggests that specimen selection must be integrated into a multimodal strategy. The discordant results highlight methodological limitations: the identification of 135 smear-negative/culture-positive cases indicates the inadequacy of smear microscopy for low-level bacterial shedding particularly in immunocompromised or extrapulmonary cases^1^. Conversely, 20 smear-positive/PCR-negative cases may be attributed to PCR inhibitors or primer limitations, similar to findings in Indonesian studies where inhibitory substances in clinical samples affected PCR accuracy^11^. Furthermore, the 17 cases that were negative by smear and culture but positive via PCR highlight the potential detection of residual DNA from inactive or past infections, necessitating rigorous clinical correlation to differentiate active disease.

The peak in TB diagnoses during spring (27.8%; 20 April–20 May) corresponds to findings in Mazandaran Province, Iran, where post-winter surges were attributed to indoor crowding and delayed care-seeking^20^. Similar patterns in Pakistan and India suggest a connection between seasonal vitamin D fluctuations, which influence immune response, and TB susceptibility^21, 22^. In Mashhad, cultural practices during Nowruz (Iranian New Year in March) may contribute to delays in diagnosis, as families prioritize celebrations over healthcare. Vitamin D deficiency, worsened by limited winter sunlight, likely compromises innate immunity during peak transmission months, leading to a “diagnostic lag” observed in spring^19^. Future studies should assess vitamin D levels throughout the year to confirm this hypothesis.

## Limitations and methodological considerations

The single-center design and retrospective data from Qaem Hospital in this study may limit its generalizability to regions with different healthcare infrastructures. A primary limitation is the reliance on laboratory-based registry data, which precluded the inclusion of individual-level clinical confounders such as HIV status, diabetes mellitus, smoking history, and BCG vaccination records. While these factors are known to influence TB susceptibility and clinical presentation, they were not systematically linked to the microbiological referral database for the large cohort of 14,572 suspects. Consequently, the observed associations with age and gender must be interpreted as descriptive of the diagnostic yield (TPR) in this specific setting rather than as adjusted risk factors. Furthermore, the exclusion of socioeconomic variables such as income and education restricts the analysis of structural determinants, despite the known links between poverty and TB incidence. Moreover, the diagnostic heterogeneity in the study, where outpatients underwent combined smear-culture testing and inpatients received smear-only screening, introduces selection bias that could potentially underestimate paucibacillary cases in hospitalized populations. The overlapping study period with the COVID-19 pandemic further complicates distinguishing long-term trends from pandemic-specific anomalies, highlighting the need for longitudinal follow-ups to disentangle these effects.

## Recommendations for future research

Future research should prioritize elucidating the biological mechanisms underpinning gender disparities in TB susceptibility within the Iranian context. Given the retrospective nature of current registry data, prospective studies are urgently needed to incorporate individual-level clinical confounders—such as diabetes mellitus, HIV status, smoking history, and BCG vaccination records—to allow for robust multivariable adjustments. Such investigations should also explore the interplay of Vitamin D deficiency, hormonal influences, and immune response modulation to validate the gendered susceptibility hypotheses.Concurrently, multicenter collaborations across high-burden regions, such as Sistan and Baluchestan, are critical to unravel geographic heterogeneity in transmission dynamics. Building on our findings regarding laboratory yield, future research should specifically link high-yield specimens like bronchoalveolar lavage (BAL) with specific clinical manifestations and radiological patterns to refine diagnostic triage in tertiary settings.

To address diagnostic bottlenecks, studies must evaluate cost-effective algorithms—such as PCR-first protocols—to streamline testing accuracy. Furthermore, genomic surveillance of Mycobacterium tuberculosis strains is essential in Mashhad to track the transmission of drug-resistant genotypes across religious and transit borders. Equally vital is exploring sociocultural determinants, including the impact of cultural practices like Nowruz on healthcare-seeking behavior, through mixed-methods approaches. Finally, pandemic preparedness frameworks should trial scalable interventions, such as decentralized community-based screening, to ensure continuity of TB services during emergencies. These multidisciplinary efforts will bridge gaps in understanding biological vulnerabilities and systemic resilience, ultimately informing equitable, evidence-based TB control strategies.

## Conclusion

This study highlights the intricate relationship between demographic, environmental, and systemic factors influencing TB diagnostic yield in Mashhad. The higher Test Positivity Rate among women and the elderly suggests the necessity of geriatric-focused screening, while acknowledging that the weak association for gender limits its use as a primary predictor. Furthermore, the high diagnostic yield of bronchoalveolar lavage underscores the critical role of specialized specimens in tertiary care. Seasonal shifts and the impact of COVID-19 disruptions expose vulnerabilities that call for integrated and flexible public health strategies. While cautious regarding extrapolation to national policies due to the single-center design, these findings allow policymakers to develop targeted interventions to reduce morbidity in Northeast Iran and similar settings. Future research should address confounding variables like comorbidities to promote equitable TB management.

## Data Availability

Data will be made available on request to Dr. Ehsan Aryan.
